# London Medical Schools
*For fuller information in regard to these hospitals see Burdett's "Hospitals and Charities, 1898."


**Published:** 1898-09-03

**Authors:** 


### The Institutional Workshop.

### LONDON MEDICAL SCHOOLS.*

### CHARING CROSS HOSPITAL MEDICAL SCHOOL.

The hospital contains 180 beds, and 2,119 in-patients have been received in the year. The hospital is now being extended; and the adjoining Royal Westminster Ophthalmic Hospital contains 30 beds for eye cases.

The composition fee is £115 10s. on entry, or £127 1s. by instalments. Sons of medical men pay £105, or £115 10s. by instalments.

The following scholarships and prizes are given:—

*Entrance Scholarships* of the value of 100 guineas, 60 guineas, 40 guineas, 55 guineas (sons of medical men), and 30 guineas (dental). One Epsom Scholarship, giving a free education, is placed at the disposal of the Royal Medical Benevolent College, to be competed for by foundation scholars who have passed the preliminary scientific examination for the London M.B. Two Universities Scholarships of the value of 60 guineas each to students of Oxford, Cambridge, and London, who have passed certain examinations, but have not entered any London Medical School.

*Other Scholarships and Prizes* of the value of over £550 are awarded annually.

The athletic ground of the Students' Club is situated at Eltham, and can be reached in half an hour from Charing Cross.

### ST. GEORGE'S HOSPITAL MEDICAL SCHOOL.

The hospital contains 351 beds, and about 4,172 in-patients are received in the year.

The composition fee is £150 in one payment, or £160 by instalments.

The following scholarship prizes, &c., are given:—

*Entrance Scholarships*—£150 (to sons of medical men), £50, £50, two of £85 (for Oxford or Cambridge men), £85 (for students from provincial university colleges).

*Other Scholarships and Prizes* are given of the following value: £100, £40, £30, £32, £32, £18, six of £10 10s. each, and various others.

### GUY'S HOSPITAL MEDICAL SCHOOL.

The hospital contains more than 600 beds, and receives over 6,600 in-patients in the year. The eye wards contain 36 beds, and the clinical wards contain 40 beds. In the latter the most interesting medical cases, which form the subjects of the clinical lectures, are collected. A few beds in the gynæcological ward are to be reserved for cases of difficult labour.

The composition fee is 150 guineas, if paid on entrance, or 160 guineas by four annual instalments.

The following scholarships, &c., are given:—

*Entrance Scholarships.*—£100, £50, £150, £60, £50.

*Other Prizes, &c.*—£20, £15, £10, £15, £5, £5, £10, £15, for three years; £10, £20, £15, £10, £34, £25, £20, £150 per annum for three years, and £31 10s. per annum for three years, besides three gold medals.

The clubs' union recreation-ground is situated at Honor Oak Park.

Facing the hospital, and connected with it by a subway, is the college. This building serves as a residential college for about 50 students, besides providing rooms for the house physicians, house surgeons, obstetric residents, and externs. It also accommodates the students' club.

### KING'S COLLEGE MEDICAL FACULTY.

The hospital contains 220 beds, receiving 2,703 in-patients in the year.

The composition fee is £135.

The following scholarships, &c., are given:—

*Entrance Scholarships.*—£75, £75, £60, £40, £20, £60, £40, 70 guineas, and 60 guineas.

*Other Prizes, &c.*, are open to matriculated students to the value of £300.

The athletic club has a recreation ground at Wormwood Scrubs.

Residential chambers are available for the use of students.

### LONDON HOSPITAL MEDICAL SCHOOL.

The hospital contains 786 beds, being thus the largest general hospital in the kingdom, and has received 11,337 in-patients in the year.

The composition fee is £126 in one payment, or £136 10s. in three annual instalments. A reduction of 15 guineas is made to sons of medical men.

The following scholarships, &c., are given:—

*Entrance Scholarships.*—£120, £60, £60, £35, £126, £30, and £20.

*Scholarships, Prizes, &c., given after entrance.*—£20, £25, £20, £10, £20, £20, £20, £10, £35, £20, and £26; and other prizes amounting to £9, to £60, and to £10.

The college is at present being enlarged, and the new laboratories and class-rooms will be completed by October 1st.

The club's union athletic ground is within easy reach of the hospital.

### LONDON SCHOOL OF MEDICINE FOR WOMEN.

The Royal Free Hospital, in connection with which this medical school is carried on, contains 170 beds. About 2,070 in-patients are received in the year.

Students can also attend the fever hospitals of the Metropolitan Asylums Board, and are admitted to the practice of a considerable number of special hospitals.

The composition fee is £125 in one payment, or £135 in four instalments.

The following scholarships, &c., are given:—

*Entrance Scholarship.*—£30.

*Other Scholarships and Prizes* are of the following value: £30, three of £20 each, £50, and many others. Some of these, however, are coupled with the condition that the recipient shall practise in India.

Both school and hospital have recently been much improved, the former especially, a large block containing new laboratories, with all modern improvements, having been opened by the Princess of Wales only last month.

* For fuller information in regard to these hospitals see Burdett's "Hospitals and Charities, 1893."

### MIDDLESEX HOSPITAL MEDICAL SCHOOL.

The hospital contains 321 beds, 35 of which are specially set apart for cases of cancer.

About 3,598 in-patients are received in the year. The composition fee is £126 in one payment, or £136 10s. in three annual instalments.

The following scholarships, &c., are given:—

*Entrance Scholarships.*—£100, £60, one of £60 to Oxford and Cambridge students, and one of £126 to a pupil of Epsom College.

*Other Scholarships and Prizes.*—£5 5s., £10 10s., £21, £25, £5 5s., £11 11s., £60, £40, £10, in addition to which there are class prizes.

New buildings will be opened next session which will give increased facilities for the practical study of the scientific branches.

There is a residential college accommodating about 30 students. Its frontage looks over the hospital garden, while its entrance is in Cleveland Street. The warden is the chaplain of the hospital.

### ST. BARTHOLOMEW'S HOSPITAL MEDICAL SCHOOL.

The hospital contains 752 beds, including 70 at the Convalescent Hospital at Swanley.

About 6,840 in-patients are received in the year.

The composition fee is £157 10s. in one sum, or £168 in four annual instalments.

The following scholarships, prizes, &c., are given:—

*Entrance Scholarships.*—£75, £75, £150, £50, £20.

*Other Scholarships, Prizes, &c.*—These are numerous, the total value of the scholarships and prizes being nearly £900 per annum. There is a residential college for students within the precincts of the hospital.

A very admirable recreation ground has recently been purchased at Winchmore Hill, which is easily accessible by rail. The laboratories of the school are well equipped.

### ST. MARY'S HOSPITAL MEDICAL SCHOOL.

The hospital contains 281 beds. and when the new wing is completed this number will be considerably increased. About 3,961 in-patients per annum are received.

The composition fee is £139 in one payment, or £144 in four annual instalments.

The following scholarships, &c., are given:—

*Entrance Scholarships* of the value of £144, two of £78 15s., £52 10s., £138 (Epsom College), two of £57 15s.

*Other Scholarships.*—Four of the value of £20 are given at the end of each summer session.

There is a residential college in connection with the medical school, in which students may reside under the supervision of the Warden.

### ST. THOMAS'S HOSPITAL MEDICAL SCHOOL.

The hospital contains 572 beds, 43 of which belong to St. Thomas's Home.

Standing, as it does, opposite the Houses of Parliament, it occupies one of the finest and most prominent positions in London. About 6,035 in-patients are received in the year.

The composition fee is £150 on entrance, or £157 10s. instalments.

The following scholarships, &c., are given:—

*Entrance Scholarships.*—£150, £60 (with a consolation prize of £20 at the discretion of the School Committee), £50 for those coming up from the Universities.

*Other Scholarships, &c.*—Scholarships and prizes amounting to a value of £300 are awarded at the sessional examinations, as well as several medals.

Many structural improvements have recently been made both in the hospital and in the school buildings.

There is no residential college, but there is a good club. A recreation-ground of more than nine acres is provided at Chiswick.

### UNIVERSITY COLLEGE MEDICAL FACULTY.

The hospital contains 185 beds, and the number of in-patients has been about 3,020 in the year. The building is at present undergoing reconstruction, a very large sum having been given by Sir Blundell Maple for the purpose, and the works have, so far, proceeded with such rapidity that it is fair to hope that by the time students now entering have passed their anatomy, and got into the wards, they will have the run of a very fine hospital indeed.

The composition fee is £141 15s. in one sum, or £147 in three annual instalments.

The following scholarships, &c., are given:—

*Entrance Scholarships.*—One of 131 guineas and two of 55 guineas.

*Other Scholarships, Prizes, &c.*, amounting to about £300, are awarded annually.

### WESTMINSTER HOSPITAL MEDICAL SCHOOL.

The hospital contains 212 beds. About 2,696 in-patients are received in the year.

The composition fee is £105 on entrance, or £115 10s. in two instalments, or £126 in six instalments.

*Scholarships, &c.*, are given during the year of the following value: £60, £40, £30, £60, £40, £40, £60, £40, £50, £50, £60, £40, £40, £60, £40, a free presentation open to pupils of Epsom College, and a Scholarship in Arts of £20 for dental students.

### COOKE'S SCHOOL OF ANATOMY, PHYSIOLOGY, AND OPERATIVE SURGERY.

Although a private institution, and unconnected with any hospital, Cooke's School must be included among the medical schools of London, among which, in fact, it fills a very useful place.

It is a private school, conducted by Mr. Cooke, F.R.C.S., which concerns itself chiefly with what may be termed supplementary work, *i.e.*, with the extra study and dissections required by those rejected at the examinations in anatomy and physiology, before they can be readmitted to examination, with special work for the higher examinations, and special dissections for the Fellowship of the Royal College of Surgeons. A strictly limited number of curriculum students are also received, not more than six being taken on in the course of the year. Curriculum, as well as supplementary, certificates from this school are accepted by the Universities and other examining bodies.

The school offers annually the Bland Sutton Presentation, named after Mr. Bland Sutton, F.R.C.S., a former pupil of the school, which confers the privilege of free education in all the subjects of the first two years of the medical curriculum. This presentation is intended to assist specially earnest and deserving men anxious to study medicine, and prevented from doing so through limited means.

